# Review of Pediatric Extraosseous Chordomas with a Unique, Illustrative Case

**DOI:** 10.1159/000528761

**Published:** 2023-01-02

**Authors:** Benjamin J. Lee, Audrey Grossen, Helen Shi, Sara Abu Mehsen, Zhongxin Yu, Kar-Ming A. Fung, Khairuddin Memon, Joanna E. Gernsback

**Affiliations:** ^a^Department of Neurosurgery, University of Oklahoma Health Sciences Center, Oklahoma City, Oklahoma, USA; ^b^Department of Pathology, University of Oklahoma Health Sciences Center, Oklahoma City, Oklahoma, USA; ^c^Department of Radiology, University of Oklahoma Health Sciences Center, Oklahoma City, Oklahoma, USA

**Keywords:** Pediatric chordoma, Extraosseous chordoma, Lumbar chordoma, Spinal oncology

## Abstract

**Introduction:**

Chordoma is a rare, aggressive tumor that is believed to originate from notochord remnants. It can occur anywhere from the clivus to the sacrum and often recurs even after resection and radiotherapy. We present a unique case that initially suggested a different pathology based on imaging and presentation but was found to be a chordoma on gross and pathological analysis.

**Case Presentation:**

An 11-year-old girl presented outpatient for scoliosis evaluation and was found to have what appeared to be a right L4 peripheral nerve sheath tumor on MRI, causing dextroconvex scoliosis. She underwent a gross total resection via a retroperitoneal approach and was found to have what appeared to be an extraosseous, extradural, extra-spinal canal lumbar chordoma. Immunohistochemical features on surgical pathology were consistent with chordoma. The patient was referred to radiation oncology for adjuvant radiotherapy and pediatric hematology/oncology for recurrence monitoring.

**Discussion:**

Our case is the first to present in such a manner, was shown to be external to the spinal canal, encasing the nerve root, and was the first such case in a pediatric patient. We reviewed the growing body of literature on spinal extraosseous chordomas and their characteristics within the pediatric patient population. We also reviewed chordoma pathogenesis theories as well as current and future treatment options.

## Established Facts

- Chordomas are malignant, aggressive tumors derived from notochord remnants that occur most commonly in the sacrococcygeal region and the clivus, with a small subset occurring in extraosseous and/or extra-axial sites.
- The diagnosis of conventional chordoma is made with brachyury immunostaining on pathology.

## Novel Insights

- Pediatric chordoma may present with back pain and scoliosis with symptoms disproportionate to CT findings.
- Pediatric chordoma may also present as an extraosseous, extradural, extra-spinal canal lumbar mass, mimicking a peripheral nerve sheath tumor.

## Introduction

Chordomas are malignant, aggressive tumors derived from notochord remnants that occur most commonly in the sacrococcygeal region and the clivus, with a small subset occurring in extraosseous and/or extra-axial sites. They are characterized histologically by the presence of brachyury [1, 2, 3, 4]. This anatomical distribution is starkly different in children. In this population, over half are located intracranially and are destroying the clivus with extradural compression; common features seen on imaging or intraoperatively [5, 6]. Chordomas account for roughly 3% of all primary bone tumors, excluding lymphoproliferative and hematogenous neoplasms, and are the most common primary malignant spine neoplasm [6, 7]. That being said, chordomas are extremely rare overall, with an incidence rate of 0.08 per 100,000 in the general population, dropping to 0.02 per 100,000 in those younger than 40 years [1, 3, 4]. Reports of chordoma in the pediatric population, especially in an extraosseous location, are scarce. However, there have previously been rare reports of extraosseous clival and spinal chordomas in the pediatric population [8, 9, 10, 11]. It is currently unclear why extraosseous chordomas form, given our limited experience. Thus far, two reported cases of an extraosseous chordoma involving the lumbar spine in the pediatric population [8, 12]. Here, we present a unique case of an extraosseous, extradural, extra-spinal canal lumbar chordoma in a pediatric patient who presented with scoliosis, followed by a review of the literature on chordoma pathogenesis, current treatments, and prognosis. Written informed consent was obtained from the patient's legal guardian for the publication of this case report and accompanying images.

## Case Report

### History

An 11-year-old Caucasian female was referred to neurosurgery for evaluation of scoliosis and a 1-year history of back pain. The pain was right-sided and radiated down the lateral aspect of her right lower extremity and was not relieved by over-the-counter medications, including acetaminophen, naproxen, and ibuprofen. She also described occasional tingling of her right toes and the feeling that her right knee would “give out” when walking. She denied experiencing any bowel and bladder dysfunction. She also denied left-sided pain and any inciting event or injury.

### Workup

**Fig. 1:**
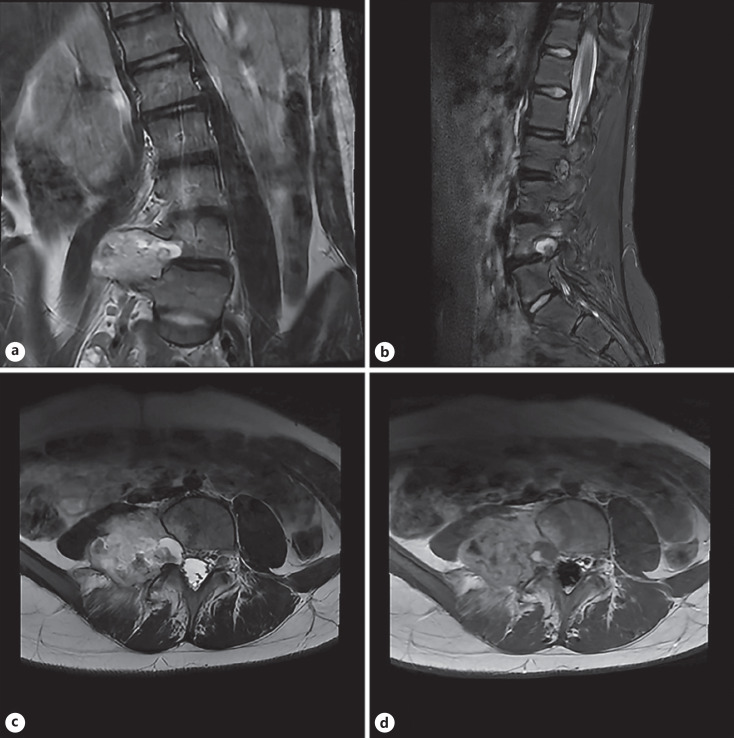
**a** Coronal T2: A T2 hyperintense mass widening the right L4-L5 neural foramen and scalloping the posterior aspect of the L4 vertebral body, extending into the right paraspinal soft tissues including the psoas musculature. **b** Sagittal STIR: there is edema in the posterior aspect of L4 vertebral body as well as the right posterior elements. **c** Axial T2: hyperintense mass widening the right L4-L5 neural foramen and scalloping the posterior aspect of the L4 vertebral body, extending into the right paraspinal soft tissues including the psoas musculature. **d** Post-contrast axial T1 shows marked enhancement in the lesion.

At the time of initial presentation, her non-contrasted CT lumbar spine and her thoracic spine X-rays showed dextroconvex scoliosis with a Cobb angle of 15°, with no other abnormalities noted by the radiologist. Given the mildness of her scoliosis, her back and the right leg pain was unlikely to be secondary to her scoliosis alone. Thus, a lumbar MRI was ordered to rule out a herniated disc or other causes of nerve impingement. Her MRI showed a 5.3 cm T2/STIR hyperintense, lobulated, heterogeneous enhancing lesion widening the right L4-L5 neuronal foramen with scalloping of the posterior right L4 vertebral body and extending into the paraspinal soft tissues, most suggestive of a right L4 peripheral nerve sheath tumor (shown in Fig. 1). There were no obvious signs of periosteal involvement.

**Fig. 2:**
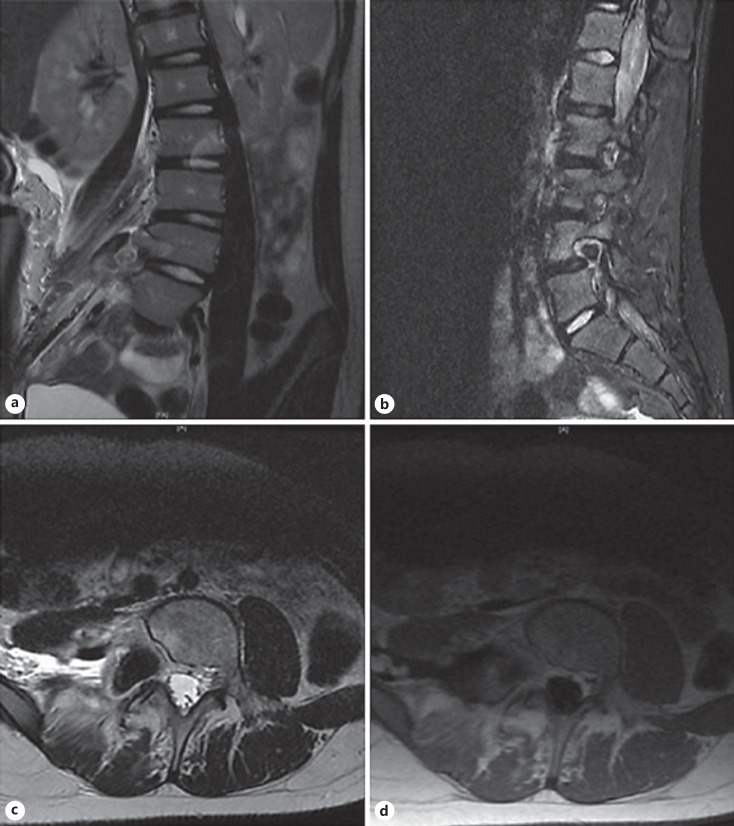
**a** Coronal T2: decompression of the right L4-L5 neural foramen, scalloping of the posterior aspect of the L4 vertebral body still present, no apparent residual tumor in the right paraspinal soft tissues and the psoas musculature is intact. **b** Sagittal STIR: slight reduction in edema in the posterior aspect of L4 vertebral body as well as the right posterior elements. **c** Axial T2: complete resection of the mass but widening the right L4-L5 neural foramen and scalloping the posterior aspect of the L4 vertebral body is still present, no residual mass seen in the right paraspinal soft tissues including the psoas musculature. **d** Post-contrast axial T1 shows no enhancement in the area where the lesion was indicating gross total resection.

The MRI findings and plan for surgical resection were discussed with the patient and her mother on follow-up. Given the lateral location of the mass, a retroperitoneal approach was planned with the assistance of pediatric surgery. Intraoperatively, we found that the mass appeared to encase the right L4 nerve root, which was spared along with any other neural elements we encountered. The mass also had a very friable capsule and contained old hematoma. We dissected the mass from the L4 nerve root with great difficulty and followed it down to the vertebral body, achieving a gross total resection (confirmed on postoperative imaging, shown in Fig. 2). The frozen sample sent intraoperatively was nondiagnostic, although chordoma was within the differential. The patient was admitted following surgery for observation and pain control; her postoperative course was uneventful. She was discharged home on postoperative day three in stable condition.

**Fig. 3:**
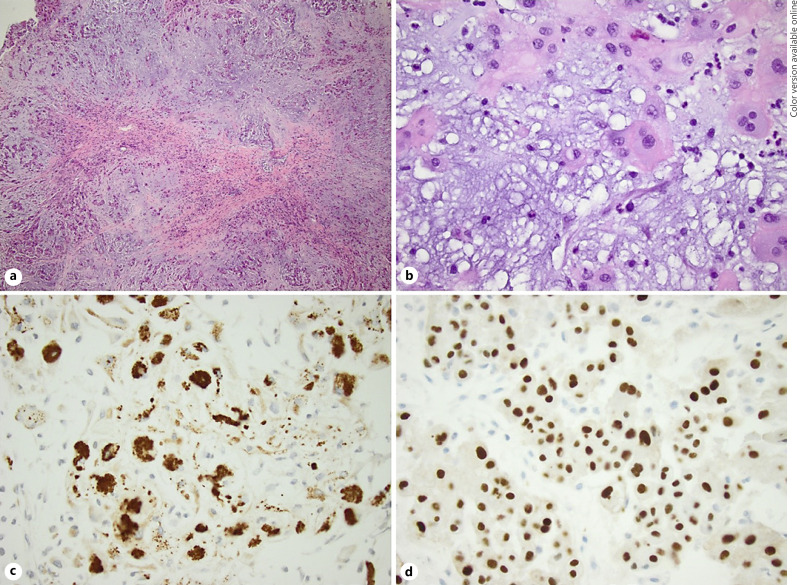
**a** Section of the tumor shows lobular pattern of short cords of eosinophilic epithelioid cells impeded in a myxoid stroma (H and E, ×40 magnification). **b** Section of the tumor highlights the physaliphorous cells along with the epithelioid tumor cells (H and E, ×400 magnification). **c** The neoplastic cells show immunoreactivity for pan-cytokeratin (×400 magnification). **d** The neoplastic cells show diffuse and strong nuclear staining for brachyury (×400 magnification).

The final pathology on the permanent sample showed that the mass was consistent with conventional-type chordoma. Histologic sections of the soft tissue mass of the right L4 vertebra show a lobular pattern with fibrous septa separating the lobules. The lobules consist of abundant basophilic myxoid stroma with short cords of eosinophilic epithelioid cells, which are admixed with vacuolated cells resembling lipoblasts and physaliphorous cells. The neoplastic cells show diffuse and strong nuclear positivity for brachyury immunostaining, immunoreactivity to pan-cytokeratin, and epithelial membrane antigen, as well as focal immunoreactivity to desmin and S-100. The tumor cells show retained nuclear staining of BRG-1 and INI-1. These features are shown in Figure 3.

Upon receiving the final pathology results, the patient was referred to pediatric hematology/oncology and radiation oncology. Adjuvant proton beam radiation was chosen to minimize acute and long-term toxicity, and the patient completed a full course. She received 74 Gy administered in 37 fractions over 51 days and routine postradiation monitoring for recurrence. She was last seen for follow-up at 3 months post-operation and reported that she has been doing well since surgery, with an improvement in her pain and weakness. However, scoliosis films at 3 months showed worsening of her curvature with a Cobb angle of 51°. Because she is still growing and undergoing radiation treatments, we referred her for bracing temporarily. We plan for a close follow-up for safe scoliosis surgery as soon as possible.

## Discussion

To our knowledge, this is the first report of a pediatric, extraosseous, extradural chordoma that was also found to be external to the spinal canal and encasing a nerve root. Extraosseous chordomas in the pediatric population are exceedingly rare, even more so for those in the lumbar spine. In a review of the literature to find similar cases, we specifically examined thoracolumbar or sacrococcygeal chordomas in the pediatric population. Of those we found (shown in Table 1), only two were extraosseous, with the patient documented in the report by Hamilton et al. having the most similarities to our patient [8, 12, 13, 14, 15, 16, 17, 18, 19, 20, 21]. In both their case and ours, the patients presented with back pain, difficulties with ambulation, radicular pain in the lower extremity, and similar postoperative treatment and course. The difference between our patient and the patient described by Hamilton et al. [12] is the tumor growth pattern that was seen on imaging and intraoperative findings. The tumor in their case appeared in the classic dumbbell pattern on imaging, which is a typical presentation of lumbosacral chordomas, and was then found to be intradural on gross dissection. In contrast, our patient's tumor was located extradurally and caused expansion of the neural foramen. It was also found invading paraspinal tissue, a behavior commonly seen in peripheral nerve sheath tumors. The diagnosis of chordoma was only made on final pathology and immunohistochemistry.

Given the findings on preoperative imaging, chordoma was low on our differential, and we counseled the patient's family on treatment for a peripheral nerve tumor. However, if we had concerns that the mass was a chordoma prior to surgery, we would have had a different conversation regarding treatment, postoperative course, and prognosis. For extraosseous chordomas, it has been shown that complete resection is more often attainable, likely due to lack of bony involvement [22, 23, 24]. While most of these reports were in cervical or intracranial extraosseous chordomas, lumbar and sacral extraosseous chordomas appear to recur less frequently [12, 24]. Since studies have shown that en bloc/GTR gives the best overall survival rate, it is important to consider extraosseous chordomas so that proper preoperative planning for GTR is done [25, 26, 27]. The combination of GTR and RT reduces the risk of recurrence, which is especially crucial in the pediatric population given their longer life span and thus, longer time periods to recur. In addition, while both conventional and proton radiotherapy allow similar target volume coverage, proton beam radiotherapy has the advantage of sparing healthy tissues close to the targeted treatment area due to protons' distal dose fall-off effect [28].

Another unique aspect of our case was that we reported histology and immunohistochemical staining results, which have yet to be reported in other pediatric extraosseous, extradural cases of chordoma. Currently, there are two theories on chordoma pathogenesis: either originate from notochord remnants or undergo a malignant transformation of a benign notochordal tumor into a chordoma [25, 29, 30, 31, 32]. However, the cause of extraosseous chordoma formation is unclear, as notochordal remnants typically stay within the spinal column. Isolated notochordal remnants have been found to escape their lineage-specific destination in the nucleus pulposus after development. Instead, they can attach to the outer surfaces of the vertebrae, where notochordal cells largely regress [33]. On histology (Fig. 3.), physaliphorous cells were present and immunohistochemical staining was positive for S-100, epithelial membrane antigen, and pathognomonic brachyury, which are all considered indicative of conventional chordoma based upon 2020 WHO Classification of Tumors of Bone: An Updated Review [2, 34, 35]. Another classification system has been proposed for vertebral chordomas based on chordoma pathogenesis, separating them based on their location and relation to the osseous structures surrounding them [36]. However, this classification has yet to be widely adopted due to the small number of cases it was based on and other cases that defy this system [23, 37]. While this system is flawed, it touches on a trend shown in multiple reports: chordomas that are extraosseous or have minimal bony involvement tend to have a better prognosis [22, 38].

With the difficulty of attaining complete remission in up to half of known cases and the adverse outcomes of repeated RT treatments, investigations into immunotherapy and chemotherapy treatments have been done. Many chemotherapeutic agents have been tried in the past but only anecdotal successes have been noted, and only Al-Rahawan et al. [39] reported success in a pediatric patient [40, 41]. Two reasons have been suggested that leads to this resistance: excessive extracellular matrix making drug delivery challenging and fewer potential therapeutic targets because of low mutational burden [42, 43]. However, epidermal growth factor receptor (EGFR) has been implicated as a potential therapeutic target [44]. Currently within the literature, there are reports of four cases of treating relapsed chordomas with the EGFR inhibitors erlotinib or cetuximab-gefitinib combination, with all four patients experiencing significant regression in disease burden with a duration of response sustained for up to a year [43, 45, 46, 47]. However, none of these studies included patients in the pediatric population, so their safety and clinical utility are unknown in this population.

## Conclusions

We present the unique case of an extraosseous, extradural, extra-spinal canal chordoma in a pediatric patient who initially presented for scoliosis and back pain. Based on imaging characteristics, the mass was initially thought to be a peripheral nerve sheath tumor, but it was confirmed to be a chordoma on surgical pathology to our surprise. The patient was treated with GTR and adjuvant RT, the primary treatment recommendation for this pathology. While extraosseous chordomas are less likely to recur and have a better prognosis, more medical treatment options are needed for this patient population. Thus far, EGFR inhibitors have shown promise, but developing other therapeutic agents is hindered by drug delivery challenges and few mutational targets.

## Statement of Ethics

The Internal Review Board of the University of Oklahoma Office of Human Research Participant Protection does not require ethics approval for case reports or series that include less than 5 patients. No ethical approval was needed in accordance with national guidelines. Written informed consent was obtained from the patient's legal guardian for publication of this case report and accompanying images. All patient information and images were de-identified before publication.

## Conflict of Interest Statement

The authors have no conflicts of interest to declare.

## Funding Sources

No funding sources were utilized in creating this manuscript.

## Author Contributions

Helen Shi, MD, and Joanna Gernsback, MD, were responsible for article conception. Helen Shi, MD, Khairuddin Memon, MD, and Joanna Gernsback, MD, were responsible for the treatment of the patient. Figures and tables were created and edited by Benjamin Lee, BS, and Audrey Grossen, BA. Literature review was performed by Benjamin Lee, BS, and Audrey Grosse, BA. Sara Abu Mehsen, MD, Zhongxin Yu, MD, and Kar-Ming A. Fung, MD, PhD, were responsible for description of pathology. Khairuddin Memon, MD, was responsible for the description and interpretation of radiological findings. Benjamin Lee, BS, and Audrey Grossen, BA, were responsible for the drafting of the article. Helen Shi, MD, Benjamin Lee, BS, and Audrey Grossen, BA, were responsible for all article revisions. Benjamin Lee, BS, Audrey Grossen, BA, Helen Shi, MD, Sara Abu Mehsen, MD, Zhongxin Yu, MD, Kar-Ming A. Fung, MD, PhD, Khairuddin Memon, MD, and Joanna Gernsback, MD, were responsible for reviewing the submitted version of manuscript. Joanna Gernsback, MD, supervised the entire process of manuscript writing, editing, and submitting.

## Data Availability Statement

All relevant data generated and analyzed during this study are included in this article. Further questions and inquiries can be directed to either the corresponding or submitting author.

## Figures and Tables

**Table 1:** Current literature on extraosseous, thoracolumbar, or sacrococcygeal chordomas in the pediatric population

Age/sex	Chordoma location	Clinical presentation	Treatment	Outcome	Author
14-mo	Multiple; C1/2, T2	Torticollis and decreased movements of the left upper limb from 3 months, an inability to sit and stand from 1 week	Spine-sparing laminectomy and decompression	Last known; on ventilator and opening eyes spontaneously with paucity of limb movement	Ramesh et al. [19]
2-yo male	T1/T2	Cold hand, trunk instability, unable to sit or stand independently	Total laminectomy from T4–T7; left costo-transversectomy of T5; received 4,500 cGy radiotherapy	Relapsed in 2 months; bilateral lower limb bone pain; left eye paralysis; osteolytic changes in the knee and shoulder	Huang, et al. [17]
9-yo male	Posterior mediastinum w/erosion into T3 vertebral body	Right-sided chest pain during URI; chest X-ray showed right posterior mediastinal mass	Complete excision; frozen showed ganglioneuroma; received 6.500 cGY of photon beam radiotherapy	Discharged 10^th^ post-op day; no issue 4 years after surgery	Ahrendt et al. [14]
4-yo	T12/L1	–	Surgically excised and local radiotherapy	Lung metastases discovered at 9 months post-op	Occhipinti et al. [13]
6-yo male	Level of L2-L3	Isolated right buttock pain and spinal rigidity of the dorso-lumbar area	Surgical resection	Not reported	Sebag et al. [8]
17-yo male	L4 w/compression of cauda equina	Several week-long history of lower left leg pain, paresthesia, and weakness	L5/L5 laminectomy followed by gross resection	Resolution of left leg paresthesia, weakness, and majority of back pain; 7 years post-op, patient showed no evidence of recurrent disease	Chau et al. [21]
11-yo male	L5-S2 w/o bone infiltration	Recent-onset lumbrosacral back pain with long-standing gait difficulties and toe walking	L4 laminoplasty 1 and L5-S1 laminectomy; resection; adjuvant proton beam therapy	At 3 months post-op, patient had recovered full strength in his lower extremity muscle groups and improvement in ambulation back to baseline. Normal bowel and bladder function throughout post-op course. Surveillance imaging up to 21 months later showed no evidence of tumor recurrence	Hamilton et al. [12]
5-yo female	Sacrococcygeal	Swelling in sacral region, constipation, urinary incontinence, and sacral pain	Radical resection of mass with transverse colostomy due to tumor invasion into colon	No follow-up	Khambekar et al. [16]
12-yo female	Sacrococcygeal	Lumbar/sacral pain; no bowel or bladder dysfunction	Negative margins; required sacrificing of S2/S3 for en bloc excision	Discharged post-op day 7; returned to active lifestyle 6 months after operation; some urinary incontinence requiring intermittent, clean catheterization, fecal incontinence due to decreased sensation	Cable et al. [15]
12-yo male	Sacrococcygeal	Swelling and pain in buttocks; no bowel/bladder dysfunction	Radical S3-S5 sacrectomy with en block abdominoperineal resection	Discharged home at 4 months post-op after complicated hospital course; started on imatinib; debulking of lung mets; 32 mo met to occipital lobe which was removed; patient alive at the time of publication	Al-Adra et al. [18]
16-yo male	Coccyx	–	–	–	Shih et al. [20]

Boldface indicates extraosseous chordoma.
